# Social Determinants of COVID-19 Vaccination Rates: A Time-Constrained Multiple Mediation Analysis

**DOI:** 10.7759/cureus.35110

**Published:** 2023-02-17

**Authors:** Kyung Hee Lee, Farrokh Alemi, Jo-Vivian Yu, Y. Alicia Hong

**Affiliations:** 1 Recreation, Parks and Leisure Services Administration, Central Michigan University, Mount Pleasant, USA; 2 Health Adminstration and Policy, George Mason University, Fairfax, USA; 3 Health Informatics, George Mason University, Fairfax, USA; 4 Health Administration and Policy, George Mason University, Fairfax, USA

**Keywords:** a chain of lasso, data-driven approach, multiple mediation analysis, social/environmental determinants, covid-19 vaccination rates

## Abstract

Objective

To estimate the multiple direct/indirect effects of social, environmental, and economic factors on COVID-19 vaccination rates (series complete) in the 3109 continental counties in the United States (U.S.).

Study design

The dependent variable was the COVID-19 vaccination rates in the U.S. (April 15, 2022). Independent variables were collected from reliable secondary data sources, including the Census and CDC. Independent variables measured at two different time frames were utilized to predict vaccination rates. The number of vaccination sites in a given county was calculated using the geographic information system (GIS) packages as of April 9, 2022. The Internet Archive (Way Back Machine) was used to look up data for historical dates.

Methods

A chain of temporally-constrained least absolute shrinkage and selection operator (LASSO) regressions was used to identify direct and indirect effects on vaccination rates. The first regression identified direct predictors of vaccination rates. Next, the direct predictors were set as response variables in subsequent regressions and regressed on variables that occurred before them. These regressions identified additional indirect predictors of vaccination. Finally, both direct and indirect variables were included in a network model.

Results

Fifteen variables directly predicted vaccination rates and explained 43% of the variation in vaccination rates in April 2022. In addition, 11 variables indirectly affected vaccination rates, and their influence on vaccination was mediated by direct factors. For example, children in poverty rate mediated the effect of (a) median household income, (b) children in single-parent homes, and (c) income inequality. For another example, median household income mediated the effect of (a) the percentage of residents under the age of 18, (b) the percentage of residents who are Asian, (c) home ownership, and (d) traffic volume in the prior year. Our findings describe not only the direct but also the indirect effect of variables.

Conclusions

A diverse set of demographics, social determinants, public health status, and provider characteristics predicted vaccination rates. Vaccination rates change systematically and are affected by the demographic composition and social determinants of illness within the county. One of the merits of our study is that it shows how the direct predictors of vaccination rates could be mediators of the effects of other variables.

## Introduction

Vaccination rates, or the percentage of a population that has received recommended vaccinations, are an important indicator of the overall health of a community. The COVID-19 pandemic has emphasized the importance of vaccination in preventing the spread of disease and protecting public health. However, vaccination rates can vary significantly within and between different populations and are often influenced by a range of social determinants [1]. Social determinants are the social and economic conditions in which people live that can impact their well-being and equity in health.

In this context, a growing body of research has examined the impact of social and environmental factors on disparities in COVID-19 vaccination rates [2]. Studies in the U.S. have found that certain groups, including those with lower levels of education, ethnic minorities, those living in remote areas, and those who are socially disadvantaged, have lower rates of vaccination, even when they have higher rates of death and transmission of disease [3,4]. Some research also found that racial/ethnic minorities and people with disability experienced lower vaccination rates than whites or non-disabled populations [5-8]. Other research investigated the associations between distinctive characteristics of neighborhood environments such as the density of vaccination sites [9], housing conditions [10], other built environments, and the incidence of COVID-19 at a state or community level in the U.S. [11]. Those findings showed that the COVID-19 pandemic has disproportionately affected Americans in socially vulnerable areas. However, previous studies heavily used cross-sectional study design to examine the associations between social/environmental determinants and vaccination rates [12,13]. Furthermore, limited longitudinal studies mainly tested a single mediation effect on the vaccination rate based on a theory-driven approach [14]. 

In contrast to a traditional approach to mediation analysis, our study uses a data-driven method to identify multiple mediators based on the timing of variable measurement. We investigated what and how these factors, directly and indirectly, affected the vaccination rates in the U.S. Specifically, we relied on an extensive set of demographics, social, economic, and neighborhood factors to examine the multiple mediation effects of social and environmental factors on vaccination rates using a longitudinal observational data.

## Materials and methods

**Figure 1:**
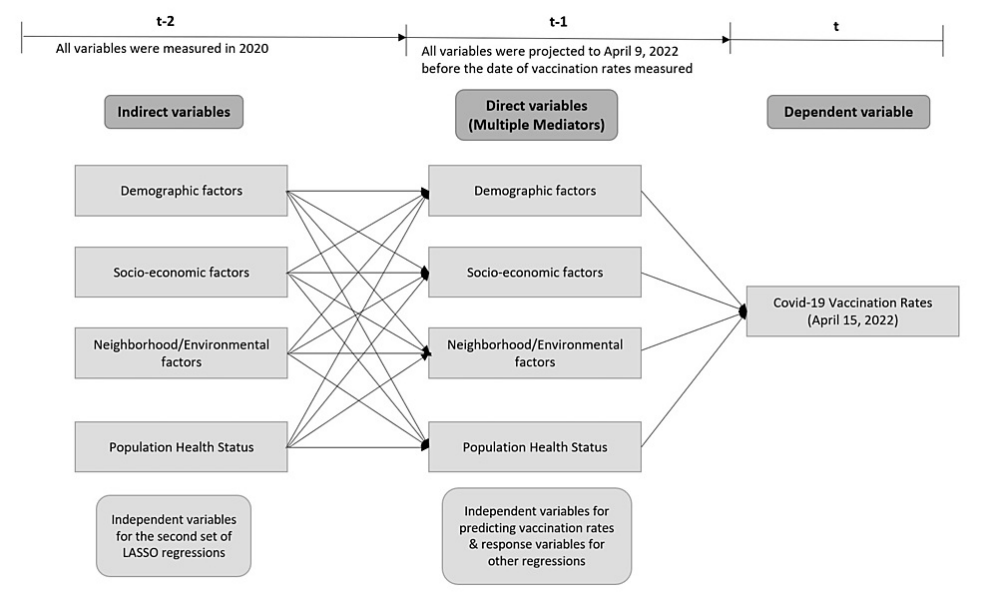
Diagram of analysis procedures LASSO: Least absolute shrinkage and selection operator

We collected data on the demographic, socioeconomic, and environmental characteristics of 3109 counties located in the continental United States that may potentially explain the variation in vaccination rates across the U.S. counties. The primary data for the percentage of the fully vaccinated population (series complete) in the counties were collected from the COVID-19 data tracker coordinated by the CDC. The number of vaccination providers per county was calculated based on the address information of provider sites using geocoding and spatial join functions. The addresses for providers at a past time frame were obtained using the Wayback Machine (https://archive.org/web/) [15]. Social, economic, and demographic factors were collected from the American Community Survey and Census Population estimate for 2019 and 2020. Additional variables were obtained from the following sources: 1) the Bureau of Labor Statistics, 2) Small Area Income and Poverty Estimates, 3) the Environmental Justice Screening and Mapping Tool, and 4) the National Center for Health Statistics. All independent variables were projected to April 2022 using measures from the two most recent years and a general linear transformation. Some of these variables were direct predictors of vaccination changes, while others were indirect or mediated predictors of vaccination rates. Direct predictors were identified through repeated least absolute shrinkage and selection operator (LASSO) regression of vaccination rates on all projected variables in April 2022. Indirect predictors were identified through repeated LASSO regression of direct predictors on independent variables measured in the prior year (Figure 1). A network model was used to depict the direct and indirect effects on vaccination rates based on two waves of data. 

This study examined the direct/indirect impact of the following 34 variables on vaccination rates: number of vaccination Providers, percentage (%) of highschool completion, % of some college education, % of unemployment, income inequality, % of children in poverty, % of children in single parent households, % of life expectancy, median household income, % of residential segregation i.e., non-White vs. White, % of traffic volume, % of home ownership, total population, % below 18 years of age, % over 65, % of non-Hispanic Black, % of American Indian or Alaska Native, % of Asian, % not proficient in English, poverty rate, % of disabilty population, % of female population, % of Hispanic population, % of Native Hawaiian Other Pacific Islander population, % of non-Hispanic Black population, % of non-Hispanic White population, % of Rural population, driving alone to work, injury death, long commute driving, premature age-adjusted mortality, residential segregation Black/White, severe housing cost burden. 

## Results

Table 1 lists all social and environmental factors with direct and indirect effects on COVID-19 vaccination rates. The second column in Table 1 reports the coefficients of robust and statistically significant predictors of vaccination rate. The LASSO regressions were repeated 10 times, and factors with robust and clinically meaningful coefficients higher than 0.05 were selected as predictors of vaccination rates in U.S. counties. Our model explained about 43% of vaccination rates in April 2022.

**Table 1:** Factors that directly, or indirectly, affect vaccination rates %: Percentage, LASSO: Least absolute shrinkage and selection operator V = % of residents fully vaccinated, A = number of vaccination providers, B = % of residents with high school completion, C = % of residents with some college, D = % of residents unemployed, E = % of children in poverty, F = % of children in single-parent households, G = life expectancy, H = median household income, I = % of residential segregation non-White vs. White, J = % of residents below 18 years of age, K = % of residents over 65 years, L  = % of residents not proficient in English, M = % of residents with disability. These variables were included in the regression analysis as independent variables, but were not statistically significant in any of the regressions: % of females, % of Hispanics, % of Native Hawaiians or other Pacific Islanders, % of non-Hispanic Blacks, % of non-Hispanic Whites, % of those living in rural areaa, % driving alone to work, number of injury/death, length of commute driving, premature age-adjusted mortality, residential segregation Black versus White, and severe housing cost burden.

	Dependent Variables in 14 LASSO Regressions													
Dependant Variable	V	A	B	C	D	E	F	G	H	I	J	K	L	M
Adjusted R Square	0.43	0.96	0.86	0.80	0.91	0.77	0.78	0.94	0.84	0.60	0.96	0.98	0.89	0.79
Prior Independent Variables														
Number of Vaccine Providers	0.10	.	.	.	.	.	.	.	.	.	.	.	.	.
% Highschool Completion	0.23	.	0.66	.	.	0.09	.	.	.	.	.	.	.	.
% Some College	0.32	.	.	0.57	.	.	.	.	.	.	.	.	.	.
% Unemployment	0.22	.	.	.	1.01	.	.	.	.	.	.	.	.	.
Income Inequality						-0.06	.	.	.	.	.	.	.	.
% Children in Poverty	-0.16	.	.	0.05	-0.08	0.78	-0.05	.	0.07	.	.	.	.	.
% Children in Single Parent Households	0.13	.	.	.	.	-0.06	0.68	.	.	.	.	.	.	.
% Life Expectancy	0.31	.	.	.	.	.	.	0.40	.	.	.	.	.	.
Median Household Income	0.13	.	.	.	.	0.17	.	.	0.97	.	.	.	.	.
% Residential Segregation Non-White Vs. White	0.09	.	.	.	.	.	.	.	.	0.42	.	.	.	.
% Traffic Volume	.	.	.	.	0.09	.	.	.	-0.05	-0.08	.	.	.	.
% Home Ownership	.	.	.	.	.	.	.	.	-0.06	.	.	.	.	.
Total Population	.	1.19	.	.	.	.	.	.	.	.	.	.	.	.
% Below 18 Years of Age	-0.25	.	.	.	.	.	.	.	-0.07	.	0.77	.	.	.
% Over 65	0.21	.	.	.	.	.	.	.	.	.	.	1.01	.	.
% Non-Hispanic Black	.	.	.	.	.	.	-0.08	.	.	.	.	.	.	.
% American Indian or Alaska Native	0.45	.	.	.	.	.	.	.	.	.	.	.	.	.
% Asian	0.24	.	.	.	.	.	.	.	-0.11	.	.	.	.	.
% Not Proficient in English	0.24	.	.	.	.	.	.	.	.	.	.	.	0.76	.
Poverty Rate	.	.	.	.	-0.09	.	.	.	.	.	.	.	.	.
% Disability Population	-0.21	.	.	.	.	.	.	.	.	.	.	.	.	0.43

The four demographics that affected vaccination rates are the percentage of residents with American Indians or Alaska Natives, Asian residents, senior residents (≥65 years), and residents with those below/who are 18 years of age. However, the percentage of Black or African American residents was not a direct predictor of vaccination rates.

The eight social determinants of illness-affected vaccination rates were the percentage of residents with high school completion, % of residents with some college, % of unemployed residents, % of children in single-parent households, median household income, % of residents living with non-White vs. White segregation, % of residents not proficient in English, % of children in poverty. As expected, the average life expectancy of residents and % of residents with disability affected vaccination rates. Notably, the higher number of vaccination providers in the county was associated with higher vaccination rates.

The following variables were included in the regression analysis as independent variables but were not statistically significant in any of the regressions: % of the female population, % of Hispanic residents, % of Native Hawaiian or other Pacific Islander residents, % of non-Hispanic Black/African American residents, % of non-Hispanic White residents, % of residents living in rural areas, % of residents driving alone to work, number of injury/death, length of commute driving, premature age-adjusted mortality, residential segregation Black versus White, and severe housing cost burden.

Table 1 also illustrates how the 15 factors that directly affected vaccination rates also mediated the effects of other factors. The first column shows factors that directly affect vaccination rates. The second to the last columns show mediated factors that indirectly affect vaccination rates. These included new variables such as the level of income inequality in the county, traffic volume, percentage of residents with home ownership, and total population.

Each column indicates one regression. The response variables listed in the first row were regressed on the independent variables listed in the first column. The explained variations in each regression are listed in the second row. Non-zero robust regression coefficients are listed in rows three to the end of the table. Blank cells indicate variables that are not predictive of the response variable.

**Figure 2:**
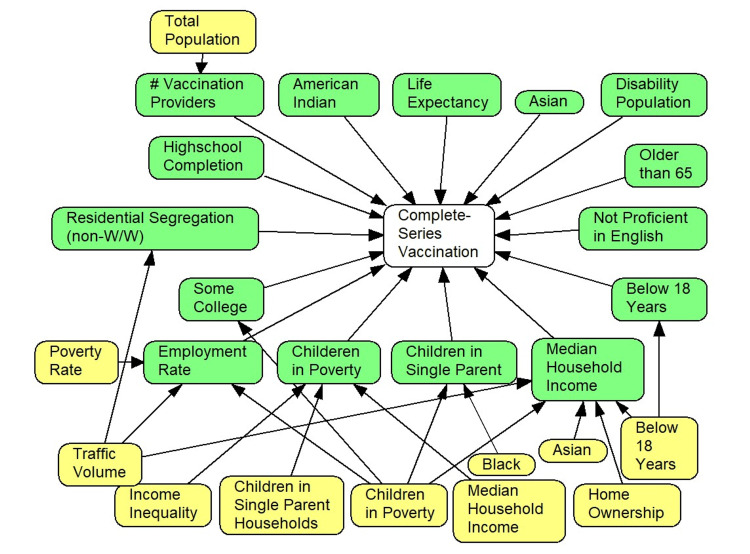
Direct and indirect predictors of vaccination rates* *Relationships between the same variable measured in two different time frames are intentionally not shown to make the network easier to understand. The network model shows variables measured at different timeframes. The outcome of interest is shown as the white-colored node, measured in April 2022. The green-colored variables were projected in April 2022 using the latest available estimations for 2019 and 2020. The yellow-colored variables were estimated in 2020. Only variables that had a non-zero (significant) relationship to vaccination rates are shown in the network. Netica (Norsys Software Corp., Vancouver, BC, Canada) estimated the probability associated with changes in one binary variable on another.

Figure 2 shows both the direct, and indirect, predictors of vaccination rate. Direct predictors are in green. Indirect predictors are in yellow. The 19 indirect predictors were all measured before April 2022. The 15 direct predictors were measured in April 2022. Some of the mediators have common causes and thus are likely to be correlated. For example, “percentage of children in poverty prior to 2022” affects “children in poverty in 2022” as well as “percentage of residents completing college in 2022” or “percentage of unemployed residents in 2022.”

## Discussion

A large percentage of the variation in the vaccination rate (43%) was explained by the factors in our model. One previous mediation study reports that 76% of the variation in vaccination rate was explained [15]. The previous study included additional variables (industry type and housing stock) besides the variables examined here. In addition, the previous study did not restrict the analysis by time of occurrence of variables; for example, demographics, social, and economic factors were measured at the same time period. Our study looked at 34 potential mediators (all occurring before vaccination rate measurement) and discovered 15 of them to be directly predictive of countywide vaccination rates. These direct predictions were also explained by 11 other indirect predictors that occurred prior to direct predictors. The point is that in our study all independent variables occur prior to the response variable. These differences in temporal constructions of the two studies can explain why one appears to be more accurate.

The findings of our study are in general agreement with the findings reported in the literature. For example, our study found that the number of vaccination providers had a moderate effect (regression coefficient of 0.10) on the county’s vaccination rate [16]. Previous studies have also shown that access to and availability of care affects vaccination rates. Our study shows that access remains a significant predictor of vaccination independent of other factors, including social determinants of vaccination rates.

A complex set of demographics were direct predictors of vaccination rates. Asian residents had higher vaccination rates than residents who were not Asian [17]. Similarly, other studies report high vaccination rates among Asians. American Indians had higher vaccination rates than non-American Indians. This finding was also reported in previous literature [18]. 

We found that African-Americans had similar vaccination rates as non-African Americans. There is support for our findings in the literature; in particular, previous studies have also shown that COVID-19 vaccine hesitancy decreased more rapidly among African Americans, and over time their vaccination rates exceeded the White population [19].

The age of the population (percentage of seniors and percentage under 18 years) also affected vaccination rates. Previous studies also confirm these relationships [20,21]. According to the CDC, teenagers and young adults aged 12 to 24 have the lowest vaccination rate among those eligible for a COVID vaccine [22]. 

A number of social determinants of illness affected vaccination rates. Previous studies confirm that vaccination rates are lower among vulnerable groups although exceptions exist [23]. Our study revealed the social determinants of vaccination. First, our study, as well as others, found that an increase in the percentage of residents with high school completion or some college, increased the vaccination rates [24]. Second, our study, and previous studies, show that single-parent households are less likely to vaccinate their children [25]. Third, this study showed that high-income counties have higher vaccination rates. A previous study also indicated that county-level income was positively associated with COVID-19 vaccination rates [26]. Fourth, counties with a higher percentage of residents with limited English proficiency had higher vaccination rates (regression coefficient 0.24). This contradicts a review of 13 published studies that showed that Hispanic immigrants have lower rates of vaccination rate [27]. Perhaps one reason is that many counties implemented interventions targeting Hispanic populations and published reports have found that residents with limited English proficiency have high vaccination rates when interventions are targeted at them [28]. Fifth, the percentage of children in poverty (and not the adult poverty rate) was a direct predictor of vaccination rates. The higher percentage of children in poverty in the county had lower vaccination rates (regression coefficient of -0.16). Other published studies also agree [29]. Sixth, this study found that increases in the unemployment rate were associated with increased vaccination rates (regression coefficient 0.22). Other published reports also indicate a positive relationship between unemployment and vaccination rates [15]. Surprisingly, counties with a higher percentage of residents living in segregated areas (White vs. Non-White but not White vs. African American) had higher vaccination rates (regression coefficient of 0.09). Some studies report that segregated African American communities have lower vaccination rates [30]. We did not find support for this claim. Also, note that we found that African American rate of vaccination was not different from non-African Americans. Finally, previous studies as well as our study show that the medical health of the population affected vaccination rates. One of the strongest predictors of vaccination rate was life expectancy: patients with higher life expectancy were more likely to receive vaccination (regression coefficient 0.31). Other studies also confirm this [31]. Counties with higher disability populations reported lower vaccination rates (regression coefficient -0.21). Others also report a negative effect of disability on vaccination rates [32]. These data suggest that the physical health of the population affects the vaccination rate.

One of the merits of our study is that it shows how the direct predictors of vaccination rates could be mediators of the effects of other variables. For example, children in poverty rate mediated the effect of (a) median household income, (b) children in single-parent homes, and (c) income inequality. For another example, median household income mediated the effect of (a) percentage of residents under the age of 18, (b) percentage of Asian residents, (c) home ownership, and (d) traffic volume in the prior year. Our findings not only describe the direct but also the indirect effect of variables.

In terms of practical implementations, our findings could provide counties with expected rates of vaccination given their characteristics. Counties can then plan programs that can change these characteristics or help them perform better than expected. The study findings would help balance the community level of health disparities; practitioners and policymakers may design and implement data-driven intervention strategies for specific residents.

There are some limitations to our study. This study is based on only two waves based i.e., 2020 and 2022. The two-year timeframe may be insufficient for observing the neighborhood or social determinant effects. Future research should be encouraged to use longer-term periods. Our study was limited in variable measurement. Results could be different if other measures are used. In our study, we did not include the effect of political factors on vaccination rates. It is possible that the inclusion of these factors could change study findings.

## Conclusions

The one certainty with the COVID-19 pandemic is that the parameters of this epidemic change over time. Some factors may have a bigger effect on the vaccination rate than others at a certain period of time. In other words, even the same factors may have a different impact on vaccination rates at different times. To reduce the potential biases in selecting variables, our study examined changes in variables over time. It was able to demonstrate how certain factors play a mediating role and how other factors indirectly affect vaccination rates. A diverse set of demographics, social determinants, and medical and provider characteristics predicted vaccination rates. In epidemiology and the social science field, mediation analysis relied on a solid theoretical foundation called confirmatory factor analysis to design the model. However, one single theory could not explain the complexity of health disparities. We demonstrated how to apply data-driven approaches to better understand the complexity by identifying direct/indirect factors on vaccination disparities across the U.S. counties. The fact that our model explained a large portion of the variation in vaccination rates suggests that we have included the appropriate variables in the model.
